# Effects of Dietary Wheat Bran on Ileal and Hindgut Digestibility of Nutrient in Pigs and Influences of Ileal Digesta Collection on Proceeding Fecal Nutrient Digestibility

**DOI:** 10.3390/ani13050799

**Published:** 2023-02-22

**Authors:** Ah Reum Son, Jeonghyeon Son, Beob Gyun Kim

**Affiliations:** Department of Animal Science, Konkuk University, Seoul 05029, Republic of Korea

**Keywords:** digestibility, fiber, hindgut fermentation, swine

## Abstract

**Simple Summary:**

The effects of dietary fiber on nutrient digestibility are relatively well-known in pigs. However, information on the influence of dietary fiber on hindgut nutrient digestibility is lacking, and ileal digesta collection before fecal collection may affect the proceeding fecal digestibility of nutrients. We determined the influences of wheat bran, a fiber-rich ingredient, on ileal digestibility, fecal digestibility, and hindgut digestibility of nutrients in pigs. Additionally, we tested the influence of ileal digesta collection on proceeding fecal nutrient digestibility. Experimental diets contained 0, 20, or 40% of wheat bran. The ileal digestibility and fecal digestibility of energy and nutrients decreased with increasing dietary wheat bran from 0 to 40%. Hindgut digestibility of dry matter and organic matter increased with increasing dietary fiber. The fecal nutrient digestibility did not differ whether fecal samples were collected before or after ileal digesta collection. Overall, the inclusion of wheat bran, a fiber-rich ingredient, reduced ileal and fecal digestibility of most nutrients but increased hindgut digestibility of some nutrients, and ileal digesta collection before fecal collection did not affect fecal nutrient digestibility.

**Abstract:**

The objectives were to determine the effects of graded inclusion rates of wheat bran (WB) on apparent ileal (AID), apparent total tract (ATTD), and hindgut digestibility of nutrients and tested the influence of ileal digesta collection on proceeding fecal nutrient digestibility in pigs. Six barrows with an initial mean body weight of 70.7 ± 5.7 kg fitted with an ileal T-cannula were used. The animals were assigned to a replicated 3 × 3 Latin square design with three diets and three periods. A basal diet was based mainly on wheat, soybean meal, and cornstarch. Two additional diets were formulated to contain 20 or 40% of WB at the expense of cornstarch. Each experimental period consisted of a seven-day adaptation period and a four-day collection period. After the adaptation period, fecal samples were collected on day 8, and ileal digesta were collected on days 9 and 10. Another set of fecal samples was collected on day 11 to determine the influence of ileal digesta collection on proceeding total tract nutrient digestibility. The AID of energy, dry matter (DM), organic matter (OM), crude protein, and phosphorus linearly decreased (*p* < 0.05) with an increasing inclusion rate of WB from 0 to 40%. The ATTD of energy, DM, OM, crude protein, ether extract, and phosphorus linearly decreased (*p* < 0.01) as the inclusion rate of WB increased. Hindgut digestibility of DM, OM, and ether extract linearly increased (*p* < 0.05) with an increasing inclusion rate of WB. The ATTD of GE and most nutrients did not differ between the two fecal collection periods of before and after ileal digesta collection. Taken together, the inclusion of a fiber-rich ingredient reduced ileal and fecal digestibility of nutrients but increased hindgut digestibility of some nutrients, and total tract digestibility of nutrients did not differ whether the fecal samples were collected before or after two days of ileal digesta collection in pigs.

## 1. Introduction

Dietary fiber in swine diets increases endogenous losses of nutrients [1] and passage rate of digesta in the gastrointestinal tract of pigs, therefore, decreases the digestibility of energy and nutrients [2,3,4]. As high-fiber ingredients are increasingly used in pig diets due to the fluctuation in the price of conventional feed ingredients [5], research on the effects of dietary fiber on the digestive physiology of pigs becomes more important for precise swine nutrition. While most nutrients are digested and absorbed in the stomach and the small intestine of pigs, a large portion of fibers is not digested and absorbed until reaching the hindgut due to the lack of endogenous fiber-degrading enzymes [6,7,8]. Consequently, the ileal digestibility of fibers is much less than that of nitrogen-free extract, protein, and fat. The influences of dietary fiber on total tract digestibility of nutrients have been investigated in many studies [4,9,10], whereas information for the hindgut digestibility (HD) of energy and nutrients is scarce due to the difficulties in determining the HD of nutrients [11,12]. Therefore, the first objective of the present study was to determine the effects of graded inclusion rates of wheat bran (WB), a high-fiber ingredient, on apparent ileal digestibility (AID), apparent total tract digestibility (ATTD), and HD of energy and nutrients in pigs.

To simultaneously determine both ileal and total tract digestibility of nutrients in pigs cannulated with a T-cannula at the distal ileum, fecal samples were collected before collecting ileal digesta [11,13,14] to avoid any potential influence of ileal digesta collection on proceeding total tract nutrient digestibility. To our knowledge, however, total tract digestibility values determined using feces collected before and after ileal digesta collection have not been compared. Thus, the second objective was to investigate the influence of ileal digesta collection before fecal collection on the ATTD of energy and nutrients in pigs.

## 2. Materials and Methods

All protocols for the experiment were reviewed and approved by the Institutional Animal Care and Use Committee of Konkuk University (KU15028; Seoul, Republic of Korea). The animal experiment was conducted in an environmentally controlled room at Konkuk University.

### 2.1. Animals, Diets, and Feeding

Six crossbred barrows with an initial body weight of 70.7 kg (standard deviation = 5.7) equipped with a T-cannula at the distal ileum were used [15]. The animals were individually housed in pens (1.2 m × 1.6 m) that were equipped with a feeder and a nipple drinker. The animals were allotted to a replicated 3 × 3 Latin square design with 3 dietary treatments and 3 periods. Potential first-order carryover effects in the Latin square design were minimized using a systemic Microsoft Excel macro developed by Kim and Stein [16].

A basal diet was prepared mainly based on wheat, soybean meal [49% crude protein (CP)], and cornstarch, and two additional diets were formulated to contain 20% or 40% of WB at the expense of cornstarch in the basal diet (Table 1 and Table 2). Soybean oil was used to maintain similar ether extract (EE) concentrations in all diets. All experimental diets contained 0.5% of chromic oxide as an indigestible index. Vitamins and minerals were included to meet or exceed the requirement estimates [17].

Daily feed allowance per pig was calculated as approximately 3% of body weight. The quantity of daily feed allowance was adjusted at the beginning of each period based on the body weight. The daily feed allowance was divided into 2 equal meals and fed to pigs at 0900 and 1700 h. Water was available at all times.

### 2.2. Sample Collection

An experimental period consisted of a seven-day adaptation period and a four-day collection period. After the adaptation period, the fecal samples were collected for 24 h from 0900 h on day 8 [18]. The ileal digesta samples were collected from 0930 to 1700 h on days 9 and 10 [19]. A plastic sample bag with wire was fixed to the T-cannula to collect the ileal digesta [20]. The plastic sample bag was changed every 30 min or when the sample bag was filled with ileal digesta. On day 11, the fecal samples were collected again for 24 h from 0900 h. Collected ileal digesta and fecal samples were immediately stored at –20 °C.

### 2.3. Chemical Analyses

The frozen ileal digesta and fecal samples were dried in a freeze drier. Samples of ingredients, diet, ileal digesta, and feces were analyzed for gross energy (GE; Parr 1261 bomb calorimeter; Parr Instruments Co., Moline, IL, USA), dry matter (DM; method 930.15, [21]), CP (method 990.03), EE (method 920.39), and amylase-treated neutral detergent fiber (aNDF; method 2002.04). Those samples were also analyzed for ash (method 942.05) to calculate organic matter (OM) and were analyzed for Ca (method 978.02) and P (method 946.06) following the AOAC [21] procedures. Chromium (Cr) concentrations in the diet, ileal digesta, and fecal samples were determined using a UV/Vis spectrophotometer (Optizen 2120UV, Mecasys Inc., Daejeon, Republic of Korea).

### 2.4. Calculations

The AID and ATTD of GE and nutrients were calculated using the following equation [22]:

AID or ATTD (%) = 100% − (Nutr_digesta_ ÷ Nutr_diet_) × (Cr_diet_ ÷ Cr_digesta_) × 100%

where Nutr_digesta_ is the concentration of GE or nutrients in the ileal or fecal digesta, Nutr_diet_ is the concentration of GE or nutrients in the experimental diet, Cr_diet_ (%) is the Cr concentration in the experimental diet, and Cr_digesta_ (%) is the Cr concentration in the ileal or fecal digesta. Units for GE and nutrients are expressed as kcal/kg and %, respectively.

The HD of GE and nutrients were calculated using the following equation [13]:

HD (%) = ATTD (%) − AID (%)

### 2.5. Statistical Analyses

Data were analyzed using the GLM procedure of SAS 9.4 (SAS Inst. Inc., Cary, NC, USA). The initial model included dietary treatment, replication, the animal within replication, and the period within replication, but only dietary treatment was used in the final model. Least squares means of each dietary treatment were calculated. Polynomial contrasts were used to test linear and quadratic effects of dietary WB on AID, ATTD, and HD of GE and nutrients. Orthogonal polynomial contrasts were used to test the effect of the fecal collection period, linear and quadratic effects of dietary WB, and interactions between the fecal collection period effect and linear and quadratic effects of dietary WB based on the 2 × 3 factorial treatment arrangement. The experimental unit was a pig. The statistical significance was declared at *p*-values less than 0.05, and the tendency at *p*-values between 0.05 and 0.10.

## 3. Results

During the experimental period, all pigs were healthy and readily consumed their feed allotments. Two observations were missing as two pigs lost their cannula during the last period.

The AID of GE, DM, OM, CP, and P linearly decreased (*p* < 0.05) with an increasing inclusion rate of WB from 0 to 40% (Table 3). The ATTD of GE, DM, OM, CP, EE, and P also linearly decreased (*p* < 0.01) as the inclusion rate of WB increased, but the ATTD of aNDF was linearly increased (*p* < 0.05). The HD of DM and OM linearly increased (*p* < 0.05) with an increasing inclusion rate of WB.

There was no interaction between the fecal collection period and dietary treatments in the ATTD of GE and nutrients (Table 4). The ATTD of GE and most nutrients did not differ between the two fecal collection periods of before and after ileal digesta collection, except that the ATTD of OM tended to be less (*p* = 0.076) when fecal samples were collected after ileal digesta collection. The ATTD of GE, DM, OM, CP, EE, and P decreased (*p* < 0.01) with increasing the inclusion rate of WB from 0 to 40% regardless of the fecal collection period.

## 4. Discussion

The chemical composition of the ingredients used in the present work (Table 1) was within a range of literature values [17]. The analyzed composition of experimental diets was reasonably similar to the expected values (Table 2). As expected, CP and aNDF concentrations in the experimental diets increased as the inclusion rate of WB increased at the expense of cornstarch.

As the experimental diets were formulated by using WB at the expense of cornstarch in the present work, the changes in energy and nutrient digestibility by the inclusion of WB would be the reflection of the digestibility differences between WB and cornstarch. Decreased AID of GE, DM, and OM is likely due to the greater nutrient digestibility in cornstarch compared with WB which contains a relatively large quantity of fibers. The AID of starch has been reported to be greater than 90% when fed to growing pigs [23,24], whereas the AID of aNDF in WB ranged from 12.5% to 33.3% in the present work. The negative effects of dietary fiber concentrations on the ileal digestibility of GE and nutrients in the present work are in good agreement with a previous study by Huang et al. [25], who reported reduced AID of GE, DM, and OM by the inclusion of WB at 0%, 9.7%, and 48.3% as a fiber source in a corn-soybean meal-based diet fed to pigs. This observation indicates that the GE, DM, and OM digestibility of WB were less than that of a corn-soybean mixed diet. The AID of GE in the basal diet observed in the present work is greater than that in the previous study [25], which is more likely due to the different basal diet formulations. Highly digestible ingredients, cornstarch and soybean oil were used at 40% and 3%, respectively, for the basal diet in the present work whereas Huang et al. [25] used corn and soybean meal that are less digestible compared with cornstarch. The larger reduction of AID of GE (84.5% to 62.5%) by increasing the WB inclusion rate in the present work compared with the data in Huang et al. [25] (66.6% to 54.7% with a WB inclusion rate of 48.3%) can be explained by the replacement of cornstarch with WB for other two diets in the present work. Similarly to the AID of GE, the reductions of AID of DM and OM by WB inclusion in the present work were also larger than those in the study by Huang et al. [25]. These observations can also be explained by the use of cornstarch in the present study whereas corn and soybean meal fed to pigs by Huang et al. [25].

The reduction of ATTD of GE, DM, and OM by increasing the inclusion rate of WB is consistent with the observations in previous experiments [25,26]. In the study by Huang et al. [25], the ATTD of GE was reduced from 83.6% to 74.1%, which is less reduction of ATTD of GE compared with the present work. Similarly to the AID, the magnitude of energy ATTD reduction is highly dependent on the differences between ATTD of WB and ATTD of ingredients used in the basal diet. The reduction of ATTD of DM and OM was more apparent compared with the previous work [25]. Jaworski et al. [26] also measured the effects of dietary WB on the ATTD of energy and nutrients, in which WB was included at 0%, 15%, and 30% in a corn-soybean meal basal diet. The ATTD of GE was reduced from 91.5% to 82.2%, which is less reduction of ATTD of GE compared with the present work. This is likely due to the different basal diets between the work by Jaworski et al. [26] and the present work and partially due to the greater inclusion rate of WB in the present work. It may be interesting to note that the aNDF concentrations in the basal diets in the present work, the study by Jaworski et al. [26], and the study by Huang et al. [25] were 4%, 9%, and 12%, respectively, which potentially explains the ATTD of GE in the basal diets. The negative correlation between aNDF and ATTD of GE has been previously reported [27]. The reason for the reduction of the ATTD of GE by increasing the inclusion rate of WB is likely due to the less ATTD of GE in WB compared with cornstarch. Apparently, over 98% of starch is digested and absorbed at the ileal level, but a large quantity of nutrients in WB is not digestible at the total tract level. The reduction of ATTD of DM and OM by the inclusion of WB would also be due to similar reasons as for the ATTD of GE.

While most ingested starch is digested and absorbed before the large intestine [23,28], the portion of undigested fibers at the ileal level becomes the substrate for microbial fermentation in the hindgut of pigs. The increased HD of DM and OM by the inclusion of WB in the present work suggests that some fibers in WB undigested at the ileal level were fermented in the hindgut and absorbed, possibly as volatile fatty acids. However, Huang et al. [25] failed to find the effects of WB inclusion on HD of DM or OM, which is likely due to the relatively high aNDF concentration (12%) of the basal corn-soybean meal diet. The fibers in corn and soybean meal may have been sufficient for microbial fermentation in the hindgut. Additionally, digesta retention time in the hindgut for microbial fermentation may have been longer in the present work, potentially due to the less fiber concentration compared with the study by Huang et al. [25], which may also be one of the reasons for the differences between the studies [29].

The linearly decreased AID and ATTD of CP by the inclusion of WB in the present work is likely due to the less CP digestibility of WB compared with wheat and soybean meal that were the sources of CP in the basal diet. The AID of CP in WB has been reported to be less than that in wheat and soybean meal [17]. Huang et al. [25] and Jaworski et al. [26] also observed decreased AID and ATTD of CP when WB replaced corn and soybean meal in the diets fed to pigs. However, the HD of CP was not affected by the inclusion of WB in the present study. Although the possibility of nitrogen disappearance from WB still exists, the present results indicate that the reductions of ATTD of CP by the inclusion of WB were mainly due to the changes in CP digestibility at the ileal level but not in the hindgut of pigs. The present observations are supported well by the results of Huang et al. [25].

In this study, to minimize potential confounding effects of EE with WB inclusion rates on nutrient digestibility, the EE concentrations were equalized in all experimental diets by lowering soybean oil inclusion rates with an increasing inclusion rate of WB as the EE concentration in the WB is greater than that in cornstarch. The reduced AID and ATTD of EE by the inclusion of WB are due to the less digestibility of EE in WB compared with soybean oil. It has been reported that free-form oils are more digestible compared with intact-form oils when fed to pigs [30]. The ATTD of EE in WB was reported to be approximately 60% [31]. The higher AID of EE than ATTD of EE resulted in negative values for the HD of EE in all experimental diets regardless of the inclusion rate of the WB. The present observation is consistent with Huang et al. [25] and Chen et al. [32] who suggested that the negative HD of EE resulted potentially from the synthesis of short-chain fatty acid by microbes in the large intestine of pigs. Additionally, it was reported that the concentration of short-chain fatty acid in the feces increased with the inclusion of WB in pig diets [33].

The lack of effects of WB on the AID of aNDF is likely due to the lack of fiber-degrading enzymes secreted in the small intestine of pigs [17] and limited concentrations of microbes in the small intestine, and the present results agree with a previous study by Huang et al. [25]. In contrast to the AID of aNDF, the ATTD of aNDF was linearly increased as the dietary WB concentrations in the present work. The reason for the increased ATTD of aNDF by the inclusion of WB is unclear. In the previous experiments [4,25,26], the ATTD of aNDF decreased by the inclusion of WB, which indicates that the ATTD of aNDF in WB was less than the ATTD of the basal diets mainly consisted of corn and soybean meal. However, the aNDF concentration of the basal diet in the present work was much less than those in previous studies [4,25,26], and thus, the contribution of microbial cell wall components to the fecal output from the pigs fed the basal diet may have been relatively large in the present work, resulting in low ATTD of aNDF. Microbial cell walls composed of peptidoglycan have been suggested to be analyzed as fiber [34], mostly aNDF [35]. Cervantes-Pahm [14] reported that pigs fed a fiber-free diet had negative values for ATTD of total dietary fiber, which indicates microbial cell wall components mostly from the hindgut of pigs are analyzed as aNDF. The contribution of microbial cell wall components to the fecal output from the pigs fed the experimental diets containing WB at 20%, or 40%, may have been reduced compared with that from the pigs fed the basal diet, resulting in increased ATTD of aNDF in the present work.

The lack of effects of dietary treatments on the AID and ATTD of Ca would be mainly due to the low Ca concentration in WB, assuming similar Ca digestibility values in limestone and dicalcium phosphate. To meet the requirement for standardized total tract digestible P concentrations among the experimental diets, the supplemental level of dicalcium phosphate decreased with an increasing inclusion rate of WB. The decreased AID and ATTD of P with an increasing inclusion rate of WB in the present work can be explained by the less digestibility of P in WB compared with dicalcium phosphate [17]. Although statistical analyses were not performed, the greater ATTD of Ca and P compared with AID values are consistent in the present work, which indicates hindgut absorption of Ca and P in pigs. In agreement, Mok et al. [13] also reported greater ATTD of P compared with AID of P. However, Zhang et al. [36] failed to find the significant hindgut digestibility of Ca and P in pigs. Potential reasons for this consistency include pig breeds, forms of dietary Ca and P, and experimental procedures. Further research is warranted to address the inconsistency in the hindgut digestibility of Ca and P.

The decreased AID and ATTD of nutrients by the inclusion of WB in the present work can be at least partially explained by the contribution of ingredient-specific endogenous losses of nutrients and the passage rate of digesta. Endogenous losses of nutrients are divided into basal endogenous losses and ingredient-specific endogenous losses [1]. In the present work, WB-specific endogenous losses are assumed to increase with the WB inclusion rate. As the quantity of the specific endogenous losses of nutrients increases, apparent nutrient digestibility values decrease due to the increased nutrient excretions. Additionally, the passage rate of digesta, although not measured in the present experiment, is also a critical factor that can affect nutrient digestibility in pigs [2,29]. An increased dietary fiber content makes the passage rate of digesta faster, and eventually, lowers the time for digestion and absorption of nutrients.

One of the hypotheses of the present work was that the nutrient digestibility values from fecal samples before ileal digesta collection and after ileal digesta collection would be different potentially due to the influence of ileal digesta collection on the hindgut environment and digesta passage. Fecal collection before ileal digesta collection is a general practice in experiments for determining both ileal and total tract digestibility in pigs [13,25,32]. In a study by Wilfart et al. [23], the ileal digesta samples were collected prior to the collecting of fecal samples, but a three-day blank period was used to minimize the potential influence of ileal digesta collection on proceeding total tract nutrient digestibility. Ileal digesta collection was assumed to lower the amount of digesta that enters the hindgut of pigs, and thus, to increase the digesta retention time for hindgut fermentation. Additionally, ileal digesta collection may also change the microbial populations in the hindgut. Thus, the potential influence of ileal digesta collection on the proceeding fecal digestibility may be dependent upon the amount of fibers that enters the hindgut of pigs [37]. Therefore, to address the hypothesis, fecal samples were collected before and after ileal digesta collection from pigs fed varying fiber concentrations in the present work. Unexpectedly, however, two days of ileal digesta collection did not affect total tract nutrient digestibility on the next day. The lack of influence of ileal digesta collection on proceeding total tract nutrient digestibility is likely due to the ileal digesta collection procedure. In the pigs equipped with an ileal T-cannula, the collected ileal digesta were not the total quantity of ileal digesta but some portion of digesta that came out of the ileum through the T-cannula. Moreover, ileal digesta were collected only for 7.5 h per day. Thus, the quantity of ileal digest that entered the hindgut may have been sufficient to maintain a general digesta retention time and microbial populations in the hindgut of pigs.

## 5. Conclusions

In conclusion, increasing the dietary aNDF concentrations from 4% to 20% reduced the digestibility of energy and nutrients but increased the hindgut digestibility of DM and OM. The total tract digestibility of nutrients did not differ whether the fecal samples were collected before or after two days of ileal digesta collection in pigs.

## Figures and Tables

**Table 1:** Analyzed energy and nutrient composition of ingredients (as-is basis) ^1^.

Item, %	Ingredient		
	Wheat	Soybean Meal	Wheat Bran
Gross energy, kcal/kg	4009	4413	4234
Dry matter	90.2	91.6	90.4
Crude protein	13.0	48.9	16.3
Ether extract	2.04	2.39	4.17
Amylase-treated neutral detergent fiber	8.27	5.40	39.64
Ash	1.64	5.46	4.42
Calcium	0.10	0.28	0.08
Phosphorus	0.33	0.66	0.95

^1^ Data are the mean of duplicate analyses of each ingredient.

**Table 2:** Ingredient, calculated, and analyzed composition of experimental diets (as-fed basis).

Item	Wheat Bran (%)		
	0	20	40
Ingredient, %			
Ground wheat	31.1	32.4	33.5
Soybean meal, 49% crude protein	23.0	23.0	23.0
Cornstarch	40.0	20.0	-
Wheat bran	-	20.0	40.0
Soybean oil	3.0	2.0	1.0
Ground limestone	0.8	1.1	1.1
Dicalcium phosphate	0.7	0.1	-
Vitamin-mineral premix ^1^	0.5	0.5	0.5
Chromic oxide	0.5	0.5	0.5
Salt	0.4	0.4	0.4
Calculated composition ^2^, %			
Crude protein	15.41	18.78	22.12
Ether extract	4.09	3.98	3.87
Amylase-treated neutral detergent fiber	3.81	11.85	19.87
Calcium	0.56	0.53	0.53
Total phosphorus	0.39	0.47	0.64
Standardized total tract digestible phosphorus	0.25	0.28	0.37
Analyzed composition ^3^, %			
Gross energy, kcal/kg	4050	4118	4080
Dry matter	92.1	90.6	90.0
Crude protein	16.1	20.1	22.0
Ether extract	4.17	4.23	4.01
Amylase-treated neutral detergent fiber	3.97	11.44	19.72
Ash	4.13	4.69	5.40
Calcium	0.69	0.63	0.68
Phosphorus	0.44	0.48	0.63

^1^ Provided the following quantities per kg of complete diet: vitamin A, 25,000 IU; vitamin D_3_, 4000 IU; vitamin E, 50 IU; vitamin K, 5.0 mg; thiamin, 4.9 mg; riboflavin, 10.0 mg; pyridoxine, 4.9 mg; vitamin B_12_, 0.06 mg; pantothenic acid, 37.5 mg; folic acid, 1.10 mg; niacin, 62 mg; biotin, 0.06 mg; Cu, 25 mg as copper sulfate; Fe, 268 mg as iron sulfate; I, 5.0 mg as potassium iodate; Mn, 125 mg as manganese sulfate; Se, 0.38 mg as sodium selenite; Zn, 313 mg as zinc oxide; butylated hydroxytoluene, 50 mg. ^2^ Calculated based on the analyzed nutrient concentrations of the ingredients and NRC (2012) values for standardized total tract digestibility of phosphorus. ^3^ Data are the mean of the duplicate analyses of each experimental diet.

**Table 3:** Apparent ileal digestibility (AID), apparent total tract digestibility (ATTD), and hindgut digestibility (HD) of gross energy and nutrients in pigs fed diets containing graded concentrations of wheat bran ^1^.

Item, %	Wheat Bran, %			SEM	*p*-Value	
	0	20	40		Linear	Quadratic
Number of observations	5	5	6			
Gross energy						
AID	84.5	75.0	62.5	1.9	<0.001	0.542
ATTD	92.1	84.8	75.7	0.6	<0.001	0.267
HD	7.6	9.8	13.2	2.3	0.108	0.842
Dry matter						
AID	82.5	70.4	56.8	1.9	<0.001	0.747
ATTD	91.1	83.4	74.2	0.7	<0.001	0.398
HD	8.6	13.0	17.4	2.4	0.021	0.988
Organic matter						
AID	85.4	75.2	62.4	1.8	<0.001	0.574
ATTD	93.7	87.0	78.8	0.5	<0.001	0.252
HD	8.3	11.8	16.4	2.1	0.016	0.844
Crude protein						
AID	78.3	77.3	68.9	2.4	0.013	0.232
ATTD	89.5	88.3	84.1	0.6	<0.001	0.082
HD	11.2	11.0	15.2	2.7	0.304	0.519
Ether extract						
AID	90.6	87.9	83.4	2.5	0.061	0.780
ATTD	80.5	68.5	53.6	6.1	0.007	0.848
HD	–10.1	–19.4	–29.8	8.2	0.108	0.956
Amylase-treated neutral detergent fiber						
AID	12.5	26.1	33.3	9.5	0.151	0.788
ATTD	32.6	40.4	42.5	2.8	0.027	0.444
HD	19.4	14.3	9.2	9.5	0.467	0.999
Calcium						
AID	25.7	22.0	27.5	5.3	0.812	0.515
ATTD	45.3	40.4	37.5	4.8	0.249	0.874
HD	19.5	18.4	10.1	3.8	0.085	0.478
Phosphorus						
AID	41.4	21.2	18.5	4.9	0.005	0.175
ATTD	45.3	31.7	21.3	4.9	0.004	0.795
HD	3.9	10.5	2.8	5.9	0.900	0.346

SEM = standard error of the mean. ^1^ Values for the ATTD and HD were calculated based on fecal samples collected before the digesta collection period (day 8).

**Table 4:** Apparent total tract digestibility of gross energy and nutrients based on fecal collection period and inclusion rates of wheat bran.

Item, %	Fecal Collection Period:	Before Ileal Collection			After Ileal Collection			RMSE	*p*-Value ^1^				
	Wheat Bran (%):	0	20	40	0	20	40		Time	Lin	Quad	T × L	T × Q
Number of observations		5	5	6	5	5	6						
Gross energy		92.1	84.8	75.7	92.1	83.3	75.3	1.5	0.270	<0.001	0.660	0.754	0.287
Dry matter		91.1	83.4	74.2	90.9	81.8	73.7	1.5	0.167	<0.001	0.864	0.849	0.271
Organic matter		93.7	87.0	78.8	93.7	85.6	78.1	1.2	0.076	<0.001	0.602	0.479	0.269
Crude protein		89.5	88.3	84.1	89.0	86.6	84.2	1.4	0.196	<0.001	0.191	0.631	0.201
Ether extract		80.5	68.5	53.6	78.0	62.1	49.7	10.3	0.253	<0.001	0.969	0.874	0.684
aNDF		32.6	40.4	42.5	38.6	32.1	38.4	6.3	0.405	0.125	0.521	0.111	0.104
Calcium		45.3	40.4	37.5	37.3	34.3	40.1	10.4	0.329	0.587	0.537	0.247	0.697
Phosphorus		45.3	31.7	21.3	37.5	26.7	26.0	10.5	0.475	<0.001	0.410	0.175	0.671

RMSE = root mean square error; aNDF = amylase-treated neutral detergent fiber. ^1^ Time = time points for fecal grab sampling; Lin, linear effect of dietary treatment; Quad, quadratic effect of dietary treatment; T × L, interaction between time points for fecal grab sampling and linear effect of dietary treatment; T × Q, interaction between time points for fecal grab sampling and quadratic effect of dietary treatment.

## Data Availability

The data presented in the current work are available.
